# New horizons in anticoagulation: Direct oral anticoagulants and their implications in oral surgery

**DOI:** 10.4317/medoral.21862

**Published:** 2017-08-16

**Authors:** Víctor Serrano-Sánchez, Jorge Ripollés-de Ramón, Luis Collado-Yurrita, Iris Vaello-Checa, Constantino Colmenero-Ruiz, Alexandra Helm, Maria-José Ciudad-Cabañas, Victoriano Serrano-Cuenca

**Affiliations:** 1 DDS. Collaborator in the Department of Medicine at the Faculty of Medicine. Faculty of Dentistry of Complutense University of Madrid; 2 DDS, PhD. Collaborator in Departament of Oral Medicine & Orofacial Surgery. Faculty of Dentistry of Complutense University of Madrid. Collaborate Professor at the Master of Oral Surgery and Implantology UC; 3 Director of the Department of Medicine. Faculty of Medicine of Complutense University of Madrid; 4 DDS. Faculty of Dentistry of Complutense University of Madrid. Collaborator in the Master in Oral Surgery at the University Hospital of Príncipe de Asturias (UAH); 5 DDS. Faculty of Dentistry of Complutense University of Madrid; 6 Department of Medicine. Faculty of Medicine of Complutense University of Madrid; 7 MD, DDS, PHD. Professor of Departament of Oral Medicine & Orofacial Surgery. Faculty of Dentistry of Complutense University of Madrid

## Abstract

**Background:**

Thrombotic disorders remain a leading cause of death in the Western World. For decades, vitamin K antagonists used in the prevention of this pathology, such as warfarin or sintrom, were the only oral agents available for long-term anticoagulation, in spite of their disadvantages.

**Material and Methods:**

An electronic database search was carried out on MedLine and The Cochrane Library Plus, without restrictions on the type of study nor dates, in English and Spanish. Abstracts were reviewed, and complete articles if necessary, considering all articles that included recommendations on DOACs and oral surgery.

**Results:**

In recent years, the so-called “new oral anticoagulants” have been introduced in clinical practice to treat those patients whose medical conditions require long-term anticoagulant treatment, replacing traditional oral anticoagulants.

**Conclusions:**

The new oral anticoagulants represent new therapeutic options, with a number of advantages such as poor interaction with food, minor drug interactions, and do not require periodic dose adjustments or routine controls. The purpose of this review is to establish an update on the new oral anticoagulants: Dabigatran, Rivarozaban, Apixaban and Edoxaban.

** Key words:**Novel oral anticoagulants, Dabigatran, Rivaroxaban, Apixaban, Edoxaban, bleeding management, oral surgery, Anti-IIa, Anti Xa.

## Introduction

Atrial fibrillation (AF) is the most frequent sustained arrhythmia in humans, affecting 1-2% of the world population. It affects 3 to 6 million people in the United States (1), whereas in Europe, it is estimated that in 2010, 8.8 million adults over the age of 55 suffered from this arrhythmia, and these figures are expected to double by 2060 (2).

The prevalence of AF increases with age, where studies report varying estimates ranging from 2% in people under 80 years of age, to 5-15% in those older than 80 (3). Therefore, AF represents a modern-day epidemic that we must face in our daily clinical practice.

For the past decades, patients with atrial fibrillation or venous thromboembolism have been managed exclusively with vitamin K antagonists. However, they possess a Narrow Therapeutic Index (NTI), where slight changes in plasma levels may lead to treatment failure (subtherapeutic concentrations) or to various adverse effects (supra-therapeutic concentrations) such as an excessive bleeding risk.

As described above, these types of drugs have some limitations, such as ongoing monitoring and dosage adjustment. In addition, there are a number of other drawbacks, such as drug and food interactions, and even with viral diseases, as well as a relatively slow onset of action (4).

Therefore, over the past few years the so-called “new oral anticoagulants”, or “direct oral anticoagulants” (DOAC) were developed. These drugs have been introduced in clinical practice to treat various diseases and medical conditions that require the use of extended anticoagulant drug therapy, such as the prophylaxis and treatment of pulmonary and venous thromboembolism, including thromboprophylaxis following orthopedic surgery; prophylaxis and treatment of thromboembolic complications associated with atrial fibrillation and/or prosthetic replacement of cardiac valves; reducing the risk of death and new thromboembolic events such as stroke or reinfarction (5).

Compared with the coumarin derivatives described above, these drugs have very specific targets in the coagulation cascade. There are currently three DOACs approved for use in the US and in several European countries, such as dabigatran etexilate (direct thrombin inhibitor), rivaroxaban and apixaban, all factor Xa inhibitors.

In addition to the three previously mentioned drugs, a fourth drug belonging to the group of factor Xa inhibitors, edoxaban, has been recently approved by the European Medicines Agency.

## Material and Methods

An electronic database search was carried out on MedLine and The Cochrane Library Plus, without restrictions on the type of study nor dates, in English and Spanish. Abstracts were reviewed, and complete articles if necessary, considering all articles that included recommendations on DOACs and oral surgery.

## New oral anticoagulants (noac)

•Dabigatran

Mechanism of action

Dabigatran etexilate (Pradaxa ®, Boehringer Ingelheim, Spain) is the first direct and reversible thrombin oral anticoagulant approved for use. It is a pro-drug that is rapidly converted, via plasma and hepatic esterases, to dabigatran. Dabigatran is a potent, direct, competitive inhibitor of thrombin (IIa Factor). Since thrombin allows the conversion of fibrinogen to fibrin in the coagulation cascade, its inhibition prevents thrombus formation. (6)

RE-LY study

The efficacy of dabigatran was first assessed by the RE-LY (7) study, a randomized, multicenter trial designed to compare two fixed doses of dabigatran with warfarin in patients who had AF and were at increased risk for stroke.

Following the results presented by the RE-LY study, we can conclude that, compared with warfarin, dabigatran administered at a dose of 110 mg has the same therapeutic effect as warfarin, but with a lower bleeding rate, whereas dabigatran administered at a dose of 150 mg has a greater therapeutic effect than warfarin in the prevention of stroke and thromboembolism, at a similar bleeding rate (8).

-Rivaroxaban

Mechanism of action

Rivaroxaban (Xarelto®, Bayer HealthCare and Johnson & Johnson Pharmaceutical Research & Development) is an oxazoline derivative, and is the first oral direct activated factor X (Xa) inhibitor to be approved for use. It is a selective, reversible and direct factor Xa inhibitor, both free and bound to the prothrombinase complex, which interferes in both the intrinsic and extrinsic pathways of the coagulation cascade (9).

ROCKET AF Studio

The efficacy of rivaroxaban compared with warfarin was assessed in the ROCKET AF (10) study, a multicenter, double-blind, randomized trial. It was concluded that rivaroxaban was noninferior to warfarin in the prevention of stroke or thromboembolism, but presented no differences in bleeding risk, although intracranial haemorrhage and fatal bleeding were reported to a lesser extent in the rivaroxaban group (11).

•Apixaban

Mechanism of action

Apixaban (Eliquis ®, Bristol-Myers Sqibb) is a potent, reversible, direct and highly selective factor Xa inhibitor. Apixaban inhibits free and clot-bound factor Xa, as well as prothrombinase activity. By inhibiting factor Xa, both thrombin formation and thrombus formation is impeded. (12)

ARISTOTLE study

The efficacy of apixaban against warfarin was assessed in the ARISTOTLE study (13), a multicenter, double-blind, randomized study. It was concluded that warfarin is highly effective in terms of stroke prevention in patients with AF, but is associated with a variable response, has drug and food interactions, requires regular monitoring and carries a risk of bleeding (including intracranial hemorrhage).

Alternative therapy with apixaban does not require monitoring and is not only more effective than warfarin in the prevention of stroke, but also provides a lower bleeding risk. In summary, in patients with AF, apixaban was superior to warfarin in the prevention of stroke and thromboembolism, and causes less bleeding, resulting in a lower death rate (14).

•Edoxaban

Mechanism of action

Edoxaban (Lixiana ® Bristol-Myers Sqibb) is a highly selective, direct and reversible factor Xa inhibitor. Edoxaban inhibits both free factor Xa and prothrombinase activity. Inhibition of factor Xa in the coagulation cascade reduces thrombin production, extends coagulation time, and reduces the risk of thrombus formation (15).

ENGAGE AF-TIMI 48 study

The efficacy of edoxaban against warfarin was assessed in the ENGAGE AF-TIMI 48 study (16). It is a multicenter, double-blind, randomized study.

It was concluded that edoxaban was noninferior to warfarin in the prevention of stroke or thromboembolism, along with a significantly lower associated risk of hemorrhage or death from cardiovascular causes (17), ( Table 1).

**Table 1:**
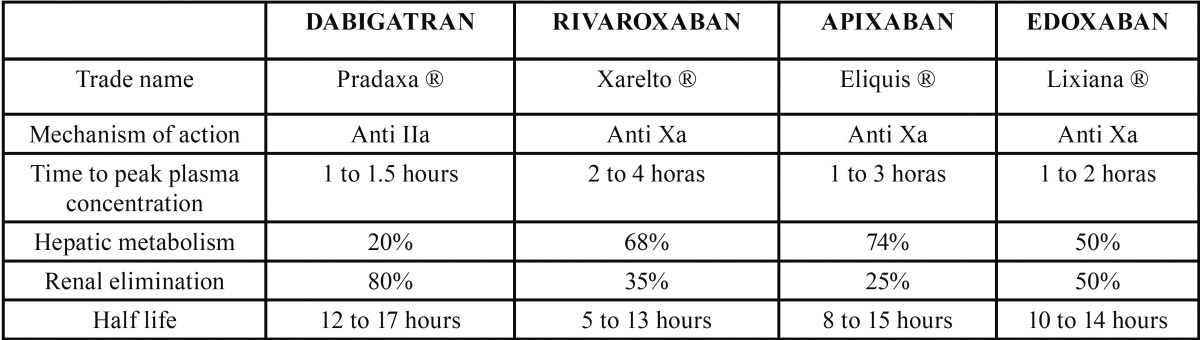
Summary of the main properties of NOACs.

## Doac interactions

DOACs are also susceptible to drug interactions. Some of the recognized interactions with the new anticoagulants are shown in  Table 2.

**Table 2:**
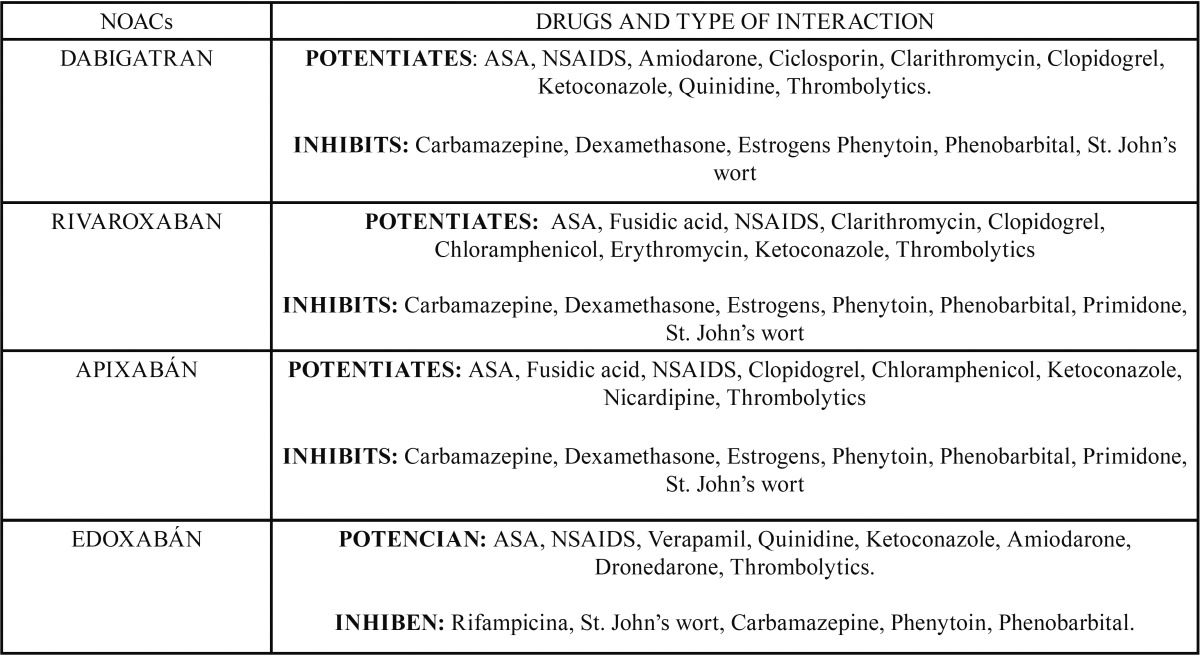
Interactions of NOACs with other drugs.

Heparins, thrombolytic and antiplatelet agents potentiate their activity and may alter haemostasis, increasing bleeding risk. All oral anticoagulants interact with each other potentiating their effects, therefore, in their management and modification, the dura-tion of their effect must be considered (18,19) ( Table 2).

## Clinical approach to DOACs

There are particular three areas of uncertainty, such as laboratory tests to monitor the degree of anticoagulation, preoperative management of the patient and management of bleeding.

-Monitoring

Although routine monitoring is not required, in certain situations it may be useful to assess the degree of anticoagulation.

Routine coagulation tests, such as INR and activated partial thromboplastin time (APTT) do not accurately reproduce the degree of anticoagulation in patients treated with DOAC. Therefore, INR is not used to monitor patients treated with direct anticoagu-lants, whereas APTT is increased in patients taking dabigatran (and more recently proven in patients treated with edoxaban), but the relationship between this increase and the received dose is yet unclear (20).

Specialized tests, including thrombin time (TT), dilute thrombin time (TTd), and ecarin time (TE) are some of the alternatives that are discussed for DOAC monitoring.

TT is a very accurate test in the assessment of dabigatran activity, and may be useful in determining when the drug has been completely cleared prior to highly aggressive surgery (21). Furthermore, TTd and TE provide the dose-response ratio that could not be determined by APTT, but these tests are not yet available in most institutions because of their high cost. Finally, Hemoclot is a TTd variant specifically calibrated for dabigatran, being a good way to identify those patients with an increased risk of bleeding (T > 60 s), which is carried out exclusively in cases of severe bleeding or urgent surgery (22).

As for rivaroxaban and apixaban, as with dabigatran, INR is also not valid, although TP and APTT can be used (through specific calibration curves). TT and TE are not useful since thrombin is not affected. A specific test that can be used in the case of factor Xa inhibitors is Heptest, which measures anti-Xa activity. Finally, in case of emergency, chromogenic methods of measuring anti-Xa activity can be used, which provide a linear dose-effect ratio (23). In the case of edoxaban, INR is not valid; TP and APTT are prolonged, but without a known link to bleeding risk; TTd is not effective; TE is unaffected, and chromogenic analysis of anti-Xa activity provides us with only quantitative data, since there are no threshold values for hemorrhage or thrombosis.

-Preoperatory management

In general, interventions with a low bleeding risk can be safely carried out without the interruption of DOAC (20). In the case of surgeries involving a moderate or severe bleeding risk, it should be weighed against the risk of thromboembolism, where the decision to suspend or delay DOAC should be individualized.

In order to objectify the risk of thromboembolism, the CHADS2 criterion (24), which classifies the general population depending on stroke risk, has been in place since 2001 ( Table 3).

**Table 3:**
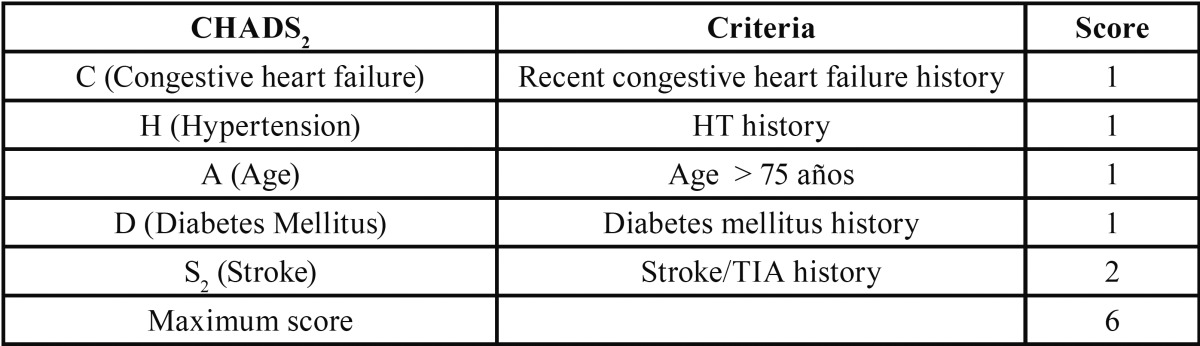
CHADS2 score for stroke risk.

However, this criterion has been modified to improve discrimination between low and moderate risk populations. Thus, a new criterion emerges in 2010, named CHA2DS2-VASc (25) ( Table 4), in which three more variables are included, such as the female sex, age range from 65 to 74 years, and cardiovascular events.

**Table 4:**
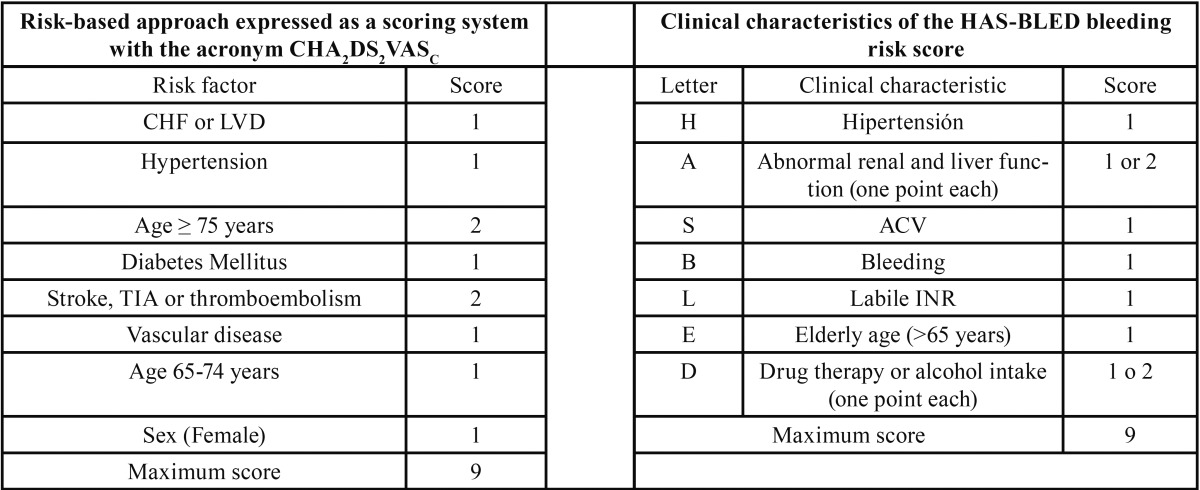
CHA2DS2VASC and HAS-BLED Scores.

Once the risk of thromboembolism has been established, the risk of bleeding should be determined. Therefore, the HAS-BLED scale has been proposed (26) ( Table 4).

**Figure 1:**
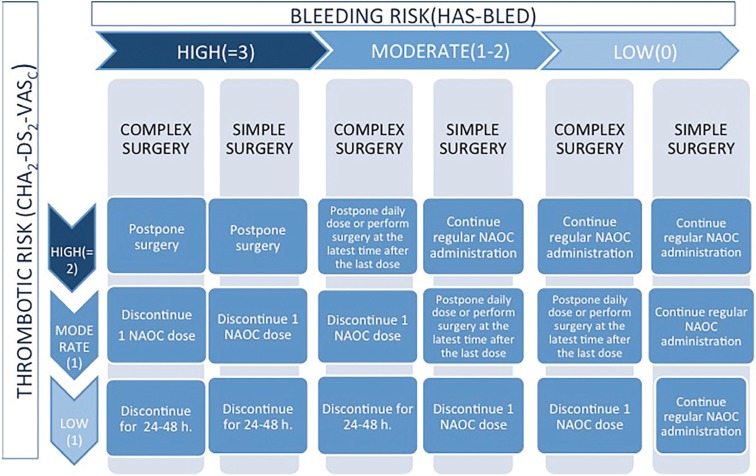
Action protocol based on bleeding and thrombotic risk. • From González Fernández-Tresguerres F. *et al.*

Once the risk of suffering a thromboembolic event and bleeding has been determined, the following guideline is generically proposed: (Fig. 1).

Discontinuation of medication prior to surgery is determined by factors such as drug half-life, specific intervention bleeding risks, and the patient’s renal function.

In general, factor Xa inhibitors should be discontinued at least 24-48 hours prior to intervention in cases of moderate bleeding risk, and 48-72 hours in cases of high risk. Dabigatran should be discontinued at least 72 hours in advance. In the case of renal dysfunction, these times should be prolonged.

Medication shall be resumed once a correct haemostasis has been achieved. In procedures with a moderate risk, it will be resumed 24 hours following the intervention, provided that correct haemostasis is achieved. In surgeries that carry a higher risk, it will be resumed 48 hours afterwards. Unlike warfarin, the onset of these drugs is very rapid, within a few hours of administration, and is therefore considered a lower risk therapy compared with vitamin K antagonists (20,27-29).

-Preoperatory management in oral surgery

The preoperative management of the patient to be surgically intervened in the oral cavity follows the previously described guidelines for direct oral anticoagulation (30-34) (Fig. 1).

Traditionally, oral surgery has been considered a type of surgery involving a low bleeding risk. However, there are certain situa-tions and interventions that imply an increased risk of bleeding, and may be considered as a moderate and even high risk.

In this manner, we can generically categorise dental treatments within three groups, depending on their relative bleeding risk: (30).

• Low risk: local anaesthetic infiltration, single dental extraction, soft tissue biopsy less than 1 cm in size, supragingival prophylaxis, placement of rubber dam, restorative procedures, crown preparation, root canal therapy, prosthetic rehabilitation of implants, and removal of orthodontic brackets and bands.

• Moderate risk: local anaesthesia nerve block, multiple simple extractions (less than 5 teeth), soft tissue biopsies ranging from 1 to 2.5 cm in size, placement of single implants, infragingival prophylaxis (6-12 teeth), and localized gingival surgery (less than 5 teeth).

• High risk: Multiple extractions of more than 5 teeth, surgical extractions requiring raising of mucoperiosteal flap and bone removal, soft tissue biopsies larger than 2.5cm in size, osseous biopsies, removal of torus, placement of multiple implants, complete periodontal treatment of all the oral cavity, gingival surgery of more than 5 teeth, endodontic surgery which involves osseous manipulation, removal of cysts and tumours, and bone regeneration procedures that involve major surgeries.

This classification is serves purely as a guideline and should be individualized according to the conditions and pathologies of each patient, as well as the specific characteristics of the intervention, for which we can use the previously described CHA2DS2VASC and HAS-BLED scales.

Once the thromboembolic and haemorrhagic risk has been assessed, a decision will be made to maintain or discontinue anticoagulant therapy (no scoring system can replace the clinician’s clinical judgment), where a consultation with the haematologist or cardiologist responsible for the patient’s medication should always be performed.

-Bleeding management

**Figure 2:**
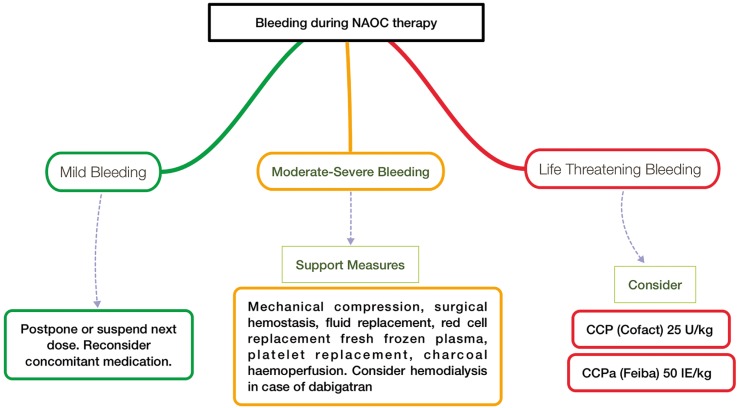
Action protocol based on bleeding risk.

Regarding the management of a bleeding event, it is important to assess whether it is life-threatening (Fig. 2). To address these events, several strategies have been proposed, such as:

• Altering the pharmacokinetics of DOACs by reducing their absorption, or carrying out their elimination. In the case of dabiga-tran and apixaban, active carbon can be administered (35). There are no specific studies on its usefulness in the case of rivaroxaban and edoxaban, although it is believed that it may also be useful. In addition, in the case of dabigatran, hemodialysis could be performed, due to its low percentage of binding to plasma proteins (36).

• Antifibrinolytic agents, such as tranexamic acid and aminocaproic acid. Their periprocedural use have proven a better haemostasis, presenting a higher safety profile than aprotinin (37).

• Plasma Factor Therapy: concentrated prothrombin complexes (CCPs), fresh frozen plasma and cryoprecipitates. The use of these factors may be helpful in the case of life-threatening bleeding, although more studies are needed to support this (38).

•Specific antidotes for each DOAC: Ciraparantag (Phase II), Andexanet Alfa (Phase II) and Idarucizumab. Currently, idaruci-zumab (Praxbind ®), approved at the end of 2015, is the only antidote to have been approved for use in the European Union, making dabigatran the first and only DOAC to have a reversing agent (38) (Fig. 2).

Regarding the measures that we must adopt before surgical interventions on the oral cavity to prevent bleeding, the following should be highlighted:

• Unless contraindicated, a local anaesthetic such as 2% lidocaine will be used with 1: 80,000 or 1:100,000 epinephrine.

• Osteotomy must be reduced to the minimum during extractions, where tooth sectioning may be indicated instead.

• Mucoperiosteal flaps should be raised as atraumatically as possible, avoiding the dissection of planes that favour a pathway for hematomas to break through.

• Thorough curettage of the cavity is essential to avoid secondary infections (an important cause of postoperative bleeding).

• As for sutures, resorbable polyglactin 910 (Vicryl ®) is preferred.

• The use of gelatin sponges, thrombin, collagen (either synthetic or porcine), and oxycellulose. Oxycellulose soaked in tranexamic acid (Kin Exogel ®) has been especially useful.

## Conclusions

• DOACs offer a safe and effective therapeutic alternative to traditional oral anticoagulants.

• Most bleeding episodes are easily controllable with local haemostatic measures.

• Before performing a dental procedure that involves the risk of associated bleeding, it is necessary to agree with the physician on the best course of action (withdrawal / non-withdrawal, or postponement of daily dose).

The authors declare no conflicts of interest.
